# Sirtuin-3: A potential target for treating several types of brain injury

**DOI:** 10.3389/fcell.2023.1154831

**Published:** 2023-03-16

**Authors:** Hongqiao Yang, Zhaopeng Zhou, Zhuanghua Liu, Junhui Chen, Yuhai Wang

**Affiliations:** Department of Neurosurgery, The 904th Hospital of PLA, School of Medicine, Anhui Medical University, Wuxi, China

**Keywords:** SIRT3, stroke, traumatic brain injury, intracerebral hemorrhage, therapeutic target

## Abstract

Sirtuin-3 (SIRT3) is responsible for maintaining mitochondrial homeostasis by deacetylating substrates in an NAD^+^-dependent manner. SIRT3, the primary deacetylase located in the mitochondria, controls cellular energy metabolism and the synthesis of essential biomolecules for cell survival. In recent years, increasing evidence has shown that SIRT3 is involved in several types of acute brain injury. In ischaemic stroke, subarachnoid haemorrhage, traumatic brain injury, and intracerebral haemorrhage, SIRT3 is closely related to mitochondrial homeostasis and with the mechanisms of pathophysiological processes such as neuroinflammation, oxidative stress, autophagy, and programmed cell death. As SIRT3 is the driver and regulator of a variety of pathophysiological processes, its molecular regulation is significant. In this paper, we review the role of SIRT3 in various types of brain injury and summarise SIRT3 molecular regulation. Numerous studies have demonstrated that SIRT3 plays a protective role in various types of brain injury. Here, we present the current research available on SIRT3 as a target for treating ischaemic stroke, subarachnoid haemorrhage, traumatic brain injury, thus highlighting the therapeutic potential of SIRT3 as a potent mediator of catastrophic brain injury. In addition, we have summarised the therapeutic drugs, compounds, natural extracts, peptides, physical stimuli, and other small molecules that may regulate SIRT3 to uncover additional brain-protective mechanisms of SIRT3, conduct further research, and provide more evidence for clinical transformation and drug development.

## 1 Introduction

The sirtuin (SIRT) family is expressed in various tissues and organs and plays different roles (Zhang T. et al., 2020; Palomer et al., 2020; Xu et al., 2021). Recently, it has gained increasing attention as a popular research target for various diseases (Carrico et al., 2018; Zhang J. et al., 2020). In the brain, it exerts a multitude of powerful functions that play an essential role in protecting the brain from various diseases and insults. It has received extensive attention in neurodegenerative diseases including Alzheimer’s disease (Ansari et al., 2017), Parkinson’s disease (Shen et al., 2020), and amyotrophic lateral sclerosis (Hor et al., 2021). Moreover, recent studies demonstrating its crucial role in several types of brain injury has sparked widespread attention.

Sirtuin-3 (SIRT3), a deacetylase located in the mitochondria, has been found in all types of human tissues and modulates energy metabolism, the respiratory chain, the production of reactive oxygen species (ROS), and autophagy *via* modification by post-translational deacetylation of the substrate (Wang et al., 2020). SIRT3 has the third highest expression at the mRNA level in adult rat brains (Cheng et al., 2016). In adult rats, SIRT3 is expressed at comparable rates at the protein level in the cerebral cortex, striatum, hippocampus, spinal cord, and brain stem, which correlates with the number of mitochondria in specific brain regions (Brandauer et al., 2015; Cheng et al., 2016). In addition to neurones, SIRT3 is expressed in astrocytes and microglia in the brain; however, at the mRNA and protein levels, it is expressed more frequently in cortical neurones than in astrocytes (Brandauer et al., 2015; Cheng et al., 2016).

Growing evidence suggests that SIRT3 may play a role in various human disorders, including cancer, metabolic diseases, and age-related illnesses (Zhang J. et al., 2020). In brain injury, SIRT3 may have functions similar to those demonstrated in other diseases. Several types of brain injury have similar pathophysiologies, such as inflammation, oxidative stress, mitochondrial dysfunction, and cell death. Mitochondria, the centres of cellular energy metabolism, play an irreplaceable role in eukaryotic life activities (Spinelli and Haigis, 2018). Mitochondrial dysfunction plays a crucial role in driving other pathophysiological events in the brain injury process, including oxidative stress, inflammation, and mitophagy (Zhang Z. et al., 2022). SIRT3 is involved in mitochondrial metabolism and homeostasis, and prevents and mitigates mitochondrial damage. Therefore, the modulation of SIRT3 is of extreme importance in the study of brain injury.

Increasing evidence has shown that SIRT3 exerts neuroprotective effects against several types of brain damage, such as cerebral ischaemia/reperfusion (I/R) injury, subarachnoid haemorrhage (SAH), traumatic brain injury (TBI), and intracerebral haemorrhage (ICH). Nevertheless, the mechanisms underlying brain protection remain unclear. It may be associated with maintaining mitochondrial homeostasis or other functions (He et al., 2020), including antioxidative stress and neuroinflammation (Gao et al., 2020), and protecting neurones against excitotoxicity (Cheng et al., 2016; Lee et al., 2021). However, the neuroprotective functions of SIRT3 in brain injury warrant further evaluation. SIRT3 may be an effective therapeutic target for alleviating brain injury. Interestingly, although most studies have shown that SIRT3 exhibits neuroprotective function against cerebral ischaemia/reperfusion damage, it has been discovered that SIRT3 may also exert a dual role.

## 2 The function of SIRT3 in brain injury

**FIGURE 1:**
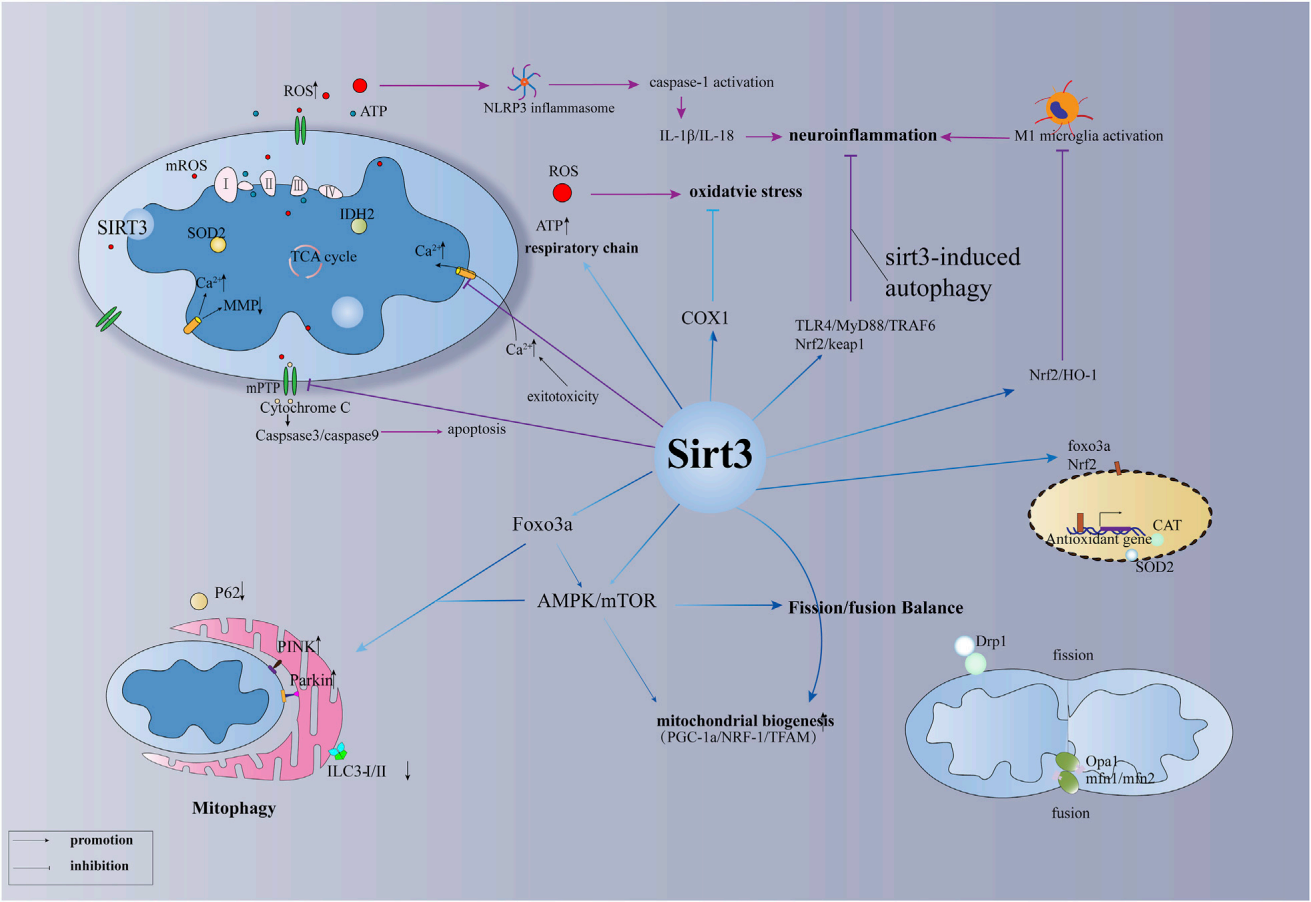
The primary function of SIRT3 in brain injury.

Here, we summarise the distinct roles of SIRT3 in several types of brain injury, involving the regulation of mitochondrial quality control and energy metabolism after brain injury as well as the regulation of oxidative stress, inflammation, and autophagy (Table 1; Figure 1). SIRT3-regulated neuroinflammation is associated with M1 microglia and pyroptosis. SIRT3 present in astrocytes and vascular endothelial cells can significantly reduce damage to the blood-brain barrier after brain injury, especially in stroke. In addition, other mechanisms associated with SIRT3, such as pyroptosis and ferroptosis, are yet to be explored. Interactions between various mechanisms, such as autophagy, inflammation, and pyroptosis, and their associated molecular networks await elucidation. When stressed, mitochondria react with other organelles in the cell, such as the nucleus and endoplasmic reticulum, to combat stress. The interaction between the mitochondrial unfolded protein response and endoplasmic reticulum may be a future focus in the study of brain damage. However, as a critical maintainer of mitochondrial homeostasis, whether SIRT3 is involved in these pathways and mechanisms remains to be further explored.

**TABLE 1:** The function of SIRT3 in brain injury.

The function of SIRT3 in brain injury					
Function	Affections	Pathways	Molecular changes	Injury styles	References
Mitochondrial function	energy metabolism	metabolic enzymes	UCP2/ATP↑ROS↓	ischemic stroke/other injury types	Dai/2014; Cimen/2010 Su/2017; Zhao/2019
		respiratory chain		TBI/other injury types	Pfleger/2015; Zhang/2022; Wang/2021
	Ca2+ overload ↓	mPTP↓	Cyt.C release/mtROS↓	TBI/other injury types	Dai/2014; Wang/2016
	mitochondrial biogenesis ↑	SIRT3/Foxo3a	PGC-1a/NRF-1/TFAM↑	SAH/ICH/other injury types	Tseng/2013; Chen/2022; zheng/2108
	mPTP↓		3CYP-D/ANT/VDAC↓	ischemic stroke/other injury types	Yang/2021; Yan/2022
	Mitophagy↑	NIX/Bnip3	ILC3/PINK1/Parkin↑ p62↓	ischemic stroke/other injury types	Dai/2017; Li/2018; Yu/2017 Yu/2020; Li/2021
		ERK-CREB			
		Bnip3			
		AMPK-mTOR			
		SIRT3/Foxo3a			
		SIRT3/AMPK			
	fusion↑/fission↓	AMPK-mTOM	Mfn1/Mfn2/OPA1↑ Drp1↓	ischemic stroke/other injury types	Samant/2014; Tseng/2013; Tyagi/2108; Zhao/2018 Klimova/2020; Hei/2022
oxidative stress		AMPK/Nrf2/SIRT3 SIRT3/PRDX-3	ROS/mtROS↓	ICH/TBI/	Gao/2018; Zheng/2018; Zhang/2022; Yang/2018
		AMPK-SIRT3 Akt-SIRT3	SOD2↑	ischemic stroke/other injury types	Brandauer/2015; Liu/2021
		SIRT3	IDH2/NADPH↑	SAH/other injury types	Chen/2022; Yu/2012;Someya/2010
		PGC-1α/ERRα/SIRT3	SOD2/ATP Synthase β↑	SAH	Zhang/2016; Zhang/2020
		SIRT3	COX-1↑SOD2↑	SAH/other injury types	Tu/2019; Zhang/2019
		AMPK-PGC-1α-SIRT3	SOD2/MMP/ATP↑		Liu/2020
		SIRT3/Foxo3a	SOD2/CAT/↑mtROS↓	ischemic stroke/other injury types	Wang/2021; Rangarajan/2015; Chang/2019; Ruankham/2021 Yang/2022
		SENP1-SIRT3	COX1/SOD2/IDH2↑	ischemic stroke	Cai/2021
		AMPK/Nrf2/SIRT3 SIRT3/AMPK/mTOR AMPK-PGC-1α-SIRT3	mtROS↓	ischemic stroke/other injury types	Gao/2018; Liu/2020; Liu/2017
inflammation	microglia activation↓	Notch/SIRT3 SIRT3/Nrf2/HO-1	Iba1+↓	ischemic stroke/ICH/other injury types	Liu/2018; Chen/2019; Liu/2021; Dai/2022; Guo/2021; Li/2022
	NLRP3 inflammasome↓	SIRT3-induced autophagy	caspase-1/IL-1β↓	other injury types	Tyagi/2018; Pi/2015; Xu/2022
			OX6/IBA1	ischemic stroke	Lee/2022
		TLR4/MyD88/TRAF6 Nrf2/keap1	NF-κBp65/TNF-a/IL-6/IL-1β/iNOS↓HO-1/NQO1/SOD/GSHpx↑ROS/MDA↓	ischemic stroke	Gao/2020
			IL-1β/IL-6/TNFα/MCP1↓	other injury types	Gao/2022; Ye/2019; Liu/2021
			NOX-2/iNOS/TNF-α↓		Liu/2018
autophagy	autophagy	SIRT3/AMPK/mTOR SIRT3-SOD2-mROS	Bcl-1/ILC3	ischemic stroke/other injury types	Dai/2017; Liu/2020; Pi/2015; Liu/2019; Chen/2021
		SIRT3		ischemic stroke	Fu/2022

### 2.1 Regulation of mitochondrial function

SIRT3 has two significant functions: mitochondrial quality control and regulation of energy metabolism. Specifically, SIRT3 is involved in the regulation of mitochondrial DNA repair, transcription, translation, fatty acid oxidation, amino acid metabolism, and the tricarboxylic acid cycle along with the regulation of the activities of the electron transport chain, ATP synthase, and related metabolic enzymes (Cimen et al., 2010; Pfleger et al., 2015; Wang et al., 2021). SIRT3 plays different roles in different types of brain injury; however, current studies on SIRT3-regulated mitochondrial function in brain injury are limited, and further investigations should be implemented into the role and mechanism of mitochondrial dynamics and mitophagy in brain injury. Here, we will focus on several downstream targets of SIRT3 that have received great attention in brain injury.

Animal experiments have shown that abnormal opening of the MPTP after ischaemic stroke leads to mitochondrial calcium overload, changes in mitochondrial membrane potential, the release of cytochrome C, and disruption of the electron respiratory chain, which leads to rapid apoptosis and irreversible brain damage (Yang Y. et al., 2021). Current research shows that three main molecules are involved in MPTP opening, CypD, VDAC1, and ANT1, which are all downstream molecules of SIRT3 (Yan et al., 2022). In particular, OPA1, acetylated by SIRT3 at K926 and K931, regulates mitochondrial dynamics, mitochondrial respiration, and mitochondrial membrane fusion and renewal (Samant et al., 2014; Chan, 2020). Importantly, PGC-1α, NRF-1, and TFAM are closely related to mitochondrial biogenesis, and PGC-1α is involved in the regulation of mitochondrial energy metabolism (Ahn et al., 2008; Dai et al., 2014). Animal experiments have demonstrated mitochondrial biogenesis regulated by SIRT3 in haemorrhagic and ischaemic stroke; however, further studies are required to confirm its role in other types of brain injury (Liu L. et al., 2021; Chen J. et al., 2022). Additionally, mitophagy plays different roles in different environments, exhibiting a two-sided role in several types of brain injury. The role of SIRT3-regulated mitophagy in brain injury should be further explored. Mitochondrial dynamics involves two aspects, mitochondrial fission and fusion, and the dynamic balance between them plays an important role in the quality control of mitochondria. Related evidence shows that the mitochondrial fission protein DRP1 and mitochondrial fusion proteins MFN1 and MFN2, regulated by SIRT3, play a vital role in brain injury, including SAH (Tyagi et al., 2018; Wu H et al., 2020). Studies have shown that excessive mitochondrial fission after brain injury has harmful effects, and promoting mitochondrial fusion is a reasonable treatment strategy. Notably, FOXO3α, an important transcription factor, is deacetylated at K271 and K290 by SIRT3, which is essential for the regulation of mitochondrial fusion/fission, mitochondrial biogenesis, and mitophagy (Tseng et al., 2013). However, the function of the SIRT3-Foxo3a pathway in brain injury requires further investigation. In addition, SIRT3 affects the mitochondrial respiratory chain and related metabolic enzymes, which in turn affect the production of ROS and ATP synthesis (Lee et al., 2022). It is well known that reducing the production of ROS and ATP following brain injury will benefit the survival of nerve cells after stress (Zheng et al., 2018). The mitochondrial respiration and energy supply involved in SIRT3 may be a promising therapeutic strategy for mitochondria-targeted therapy after brain injury.

### 2.2 Regulation of oxidative stress

Under oxidative stress, SIRT3 is essential for the regulation of the expression of mitochondrial antioxidant enzymes (Gao et al., 2018b). IDH2 is required for SIRT3 to function as an antioxidant and to defend cells against ROS. Calorie restriction stimulates increased SIRT3 to deacetylate IDH2. (Yu et al., 2012). In addition, increased SOD2 activity enhances SIRT3-dependent neuronal resistance to oxidative stress and promotes the elimination of ROS, enabling cells to instantly transition from excitement to rest (Zhang et al., 2016). Further, SIRT3-SOD2 can also resist mitochondrial oxidative stress, resulting in osteoblast differentiation and bone formation (Gao et al., 2018a). In addition, COX-1 has been confirmed as a downstream target for SIRT3 to defend against the threat of apoptosis and oxidative stress in cerebral I/R injury and H_2_O_2_-induced neuronal damage (Tu et al., 2019). Whether the SIRT3-COX1 pathway plays the same role in other types of brain damage is unknown. Since mitochondria are the primary source of ROS, SIRT3 not only controls mitochondrial respiration to decrease ROS generation but also hastens ROS removal by increasing the level of intracellular antioxidant enzymes. FOXO3α, a mitochondrion-localised transcription factor, enhances the ability of SIRT3 to resist superoxide anions by directly interacting with it (Jacobs et al., 2008). Deacetylated FOXO3α can enter the nucleus and significantly increase the expression of antioxidant enzymes CAT and MnSOD to accelerate the clearance of ROS and promote the balance of intracellular redox levels (Zhou et al., 2021). Instead of directly affecting ROS-related enzymes, the deacetylation of FOXO3 alters the activity of oxidative stress-related molecules (such as SOD2 and IDH2) in terms of molecular transcription by lowering the phosphorylation and ubiquitination of FOXO3α to maintain its structure and function (Tseng et al., 2014; Zhou et al., 2021). Additionally, SIRT3-FOXO3α regulates oxidative stress through a cascade of pathways (Rangarajan et al., 2015; Chang et al., 2019); however, the role of the SIRT3-FOXO3α pathway in brain injury remains unclear.

### 2.3 Regulation of inflammation

The activation of microglia plays an important role in neuroinflammation, which plays an important role in brain injury, especially in TBI (Karve et al., 2016). In addition, a cascade of SIRT3 pathways plays an essential role in microglial activation following brain injury (Liu S. J. et al., 2018). Recent evidence has shown that SIRT3-targeted therapy can reduce postoperative cognitive impairment by reducing microglial activation and neuroinflammation in the hippocampus (Ye et al., 2019; Liu Q. et al., 2021). The release of proinflammatory cytokines aggravates damage to the blood-brain barrier, accompanied by neuronal apoptosis, resulting in irreversible neurological damage after SAH (Fan et al., 2017; Chen J. et al., 2022). According to *in vivo* and *in vitro* studies, SIRT3 deletion can worsen brain inflammation and blood-brain barrier damage after ischaemic stroke. Yang et al. reported that reversing SIRT3 expression during the acute phase may be a promising strategy for stroke therapy (Yang X. et al., 2021). Evidence suggests that intermittent fasting can decrease microglial activation and neuroinflammation following intracerebral haemorrhage in a SIRT3-dependent manner (Dai et al., 2022). Inflammation and apoptosis play a vital role in neurological defects after subarachnoid haemorrhage, while melatonin essentially exhibits anti-inflammatory and anti-apoptotic roles by increasing the expression of SIRT3 to alleviate early brain injury after subarachnoid haemorrhage (Yang S. et al., 2018). Similarly, puerarin, a phytoestrogen extracted from Pueraria plants, also shows anti-inflammatory and anti-apoptotic effects in early brain injury after subarachnoid haemorrhage by increasing the expression of SIRT3 (Zhang Y. et al., 2019). In hypoxic-ischaemic brain injury, gastrodin inhibits microglial activation through the SIRT3 pathway and reduces inflammation in the brain (Guo et al., 2021). Triggering receptor expressed on myeloid cells 2 (TREM2) regulates the *de novo* synthesis pathway of NAD^+^, increases the level of SIRT3, and reduces the expression of NLRP3 inflammasome and caspase-1, whereas 3-TYP, an antagonist of SIRT3, can partially block the antioxidant and anti-inflammatory effects of TREM2 (Li et al., 2022). However, the mechanism by which SIRT3 reduces the expression of NLRP3 inflammasome needs further clarification. Evidence suggests that the SIRT3-SOD2 pathway is critical for the activation of the NLRP3 inflammasome following brain damage (Zheng et al., 2018), but the function of the SIRT3-Foxo3α pathway in this process is still unknown. Studies have shown that deacetylated FOXO3α can mediate crosstalk between the nucleus and plasma to bind to the TXNIP promoter by competing with chREBP (which promotes TXNIP expression) and downregulating TXNIP transcription (Tsubaki et al., 2020). The generation of ROS and TXNIP can further boost the activity of NLRP3 inflammasome, which can induce pyroptosis (Ye et al., 2017; Xu et al., 2019). Therefore, whether pyroptosis in brain injuries is affected by SIRT3 regulation requires further investigation.

### 2.4 Regulation of autophagy

It has been discovered that melatonin-mediated autophagy suppression protects against oxidative stress by involving the SIRT3-SOD2 signal pathway (Pi et al., 2015). SIRT3-SOD2-mROS, a related anti-autophagy defence system, has also been found in the photodynamic treatment of scars (Liu T. et al., 2019). By controlling the degree of acetylation of autophagy-related protein 5 (ATG5), SIRT3 has been shown to correct dysfunctional autophagy; however, the precise mechanism remains unknown (Liu P. et al., 2018). SIRT3 has various roles in autophagy and functions as a route molecule. Notably, SIRT3-AMPK-mTOR drives autophagy (Dai et al., 2017), SIRT3-ERK-CREB-Bnip3 links mitophagy (Li et al., 2018) and SIRT3-FOXO3-Parkin mediates mitophagy (Yu et al., 2017). Currently, the function and mechanism of SIRT3-mediated autophagy urgently requires further investigation in neuronal cells of the central nervous system. Mitophagy induced by SIRT3 was demonstrated to play an important role in ameliorating hippocampal injury and cognitive dysfunction (Yu et al., 2020). Luteolin, a flavonoid with diverse biological functions, has been demonstrated to protect against CIRI, which is probably associated with the SIRT3-AMPK-mTOR signalling pathway involvement in mitochondrial function rather than autophagy (Liu S. et al., 2020). Recent *in vivo* and *in vitro* experiments have demonstrated that SIRT3-mediated autophagy plays an important protective role in ischaemic brain injury (Dai et al., 2017; Chen D. et al., 2021; Fu et al., 2022). However, it is unclear whether this role could affect autophagy in the process of other types of brain injury, which warrants further discussion and research. Previous studies have shown that autophagy inhibits the production of ROS and the activation of NLRP3 inflammation, which is strongly associated with neuronal apoptosis and pyroptosis (Cao et al., 2017; Xu et al., 2022). Whether SIRT3-Foxo3a-induced autophagy can produce the same effect in brain injuries remains unknown.

## 3 The molecular regulation of SIRT3

### 3.1 Pre-transcriptional regulation

**FIGURE 2:**
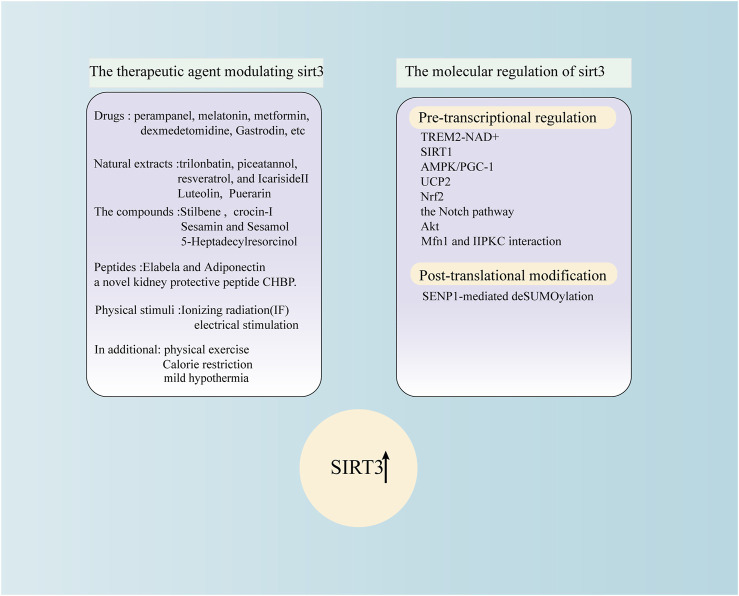
The therapeutic agent modulating SIRT3 and the molecular regulation of SIRT3.

Many factors can modulate SIRT3 at the translational and post-translational levels (Figure 2). SIRT3, as an NAD^+^-dependent deacetylase that directly supplements NAD^+^ or regulates the level of NAD^+^, is an excellent way to restore SIRT3 deacetylation activity (Klimova et al., 2020). SIRT1 and SIRT3 are crucial members of the sirtuin family. Previous research has shown that SIRT1 controls SIRT3 expression after OGD by regulating the AMPK-PGC-1α pathway (Chen et al., 2018). The SIRT1-SIRT3 axis may play an essential role in regulating the physiology of the blood-brain barrier. This may serve as a therapeutic strategy for ischaemic stroke by reducing the amount of mitochondrial ROS. Spinal cord neurones are protected from ischaemia by ZL006, a small-molecule inhibitor of the PSD-95/nNOS connection, which lowers mitochondrial oxidative stress and prevents apoptosis *via* the AMPK/PGC-1α/SIRT3 pathway (Liu et al., 2017). Similarly, *via* the AMPK/PGC-1α/SIRT3 pathway, treatment with intermittent hypoxia in cerebral ischaemia rats promotes mitochondrial biogenesis, improves mitochondrial function, repairs mitochondrial ultrastructural damage, lessens brain damage, and aids in the recovery of motor function (Su et al., 2022). In addition, the AMPK-PGC-1α-SIRT3 pathway plays an essential role in TBI brain injury (Zhang S. et al., 2022). Uncoupling protein 2 (UCP2) modifies the mitochondrial respiratory chain to regulate ATP and ROS synthesis. Researchers have suggested that UCP2 can maintain mitochondrial homeostasis and play a protective role by modulating the activity of SIRT3 by sensing energy levels (Su et al., 2017). Genipin protects against brain ischaemia-reperfusion injury by controlling the UCP2-SIRT3 signalling pathway (Zhao et al., 2019). According to experimental results, the UCP2-SIRT3-PGC-1α axis regulates adaptive mitochondrial reprogramming in the cortex following I/R injury (Meng et al., 2022). Nrf2 remains upstream of SIRT3 and controls the expression of SIRT3 (Kim et al., 2022). The entry of Nrf2 into the nucleus can promote the transcription of SIRT3 and other antioxidant genes; therefore, Nrf2 is generally regarded as a key factor in intracellular antioxidant stress (Wu et al., 2014; Diao et al., 2020). Further, it has been reported that SIRT3 is downstream of the Notch pathway (Guo et al., 2021). Notably, melatonin also elevates the transcription factors associated with mitochondrial biogenesis and activates protein kinase B (Akt)/sirtuin 3 (SIRT3)/superoxide dismutase 2 (SOD2) signalling (Liu L. et al., 2021).

### 3.2 Post-translational modification

In addition to SIRT3 regulation at the transcriptional level, post-transcriptional chemical modifications play an important role. Wang et al. have shown that during fasting, the level of SENP1 protein in mitochondria increases, which promotes fatty acid oxidation and energy consumption by deSUMOylation of SIRT3, leading to deacetylation of mitochondrial proteins (Wang et al., 2019). A recent finding suggested that SENP1-mediated deSUMOylation of SIRT3 plays an essential role in SI-AC-induced cerebral protection against I/R injury (Cai et al., 2021). At present, most studies have focused on the role of SIRT3 transcription or activity in several types of brain injury. In contrast, the regulatory role of the post-translational modification of SIRT3 in brain diseases warrants further exploration. Importantly, as a therapeutic target for a variety of diseases, the regulation of transcription and translation and post-translational modification of sirt3 are particularly important and should be further explored, and the regulation of sirt3 in other diseases needs to be further found. In addition, although SIRT1 has been shown to affect the expression of SIRT3, it is still unclear whether other members of the SIRT family affect changes in SIRT3.

## 4 The role of SIRT3 in I/R brain injury

The role of SIRT3 in ischaemic brain injury has been studied extensively compared with other types of brain injury. SIRT3 performs a dual function in ischaemic stroke as demonstrated in the current study. Initially, evidence suggested that the deacetylation activity of SIRT3 increases after stroke (Novgorodov et al., 2016). However, a recent study showed a decrease in the expression and activity of SIRT3 in the injured area (Fan et al., 2021). The experimental data showed that deletion of the SIRT3 gene reduced brain damage and deacetylation of SIRT3-dependent ceramide synthetase increasing ceramide. An increase in ceramide inhibits complex III in the respiratory chain, resulting in an increase in ROS and brain damage after ischaemia/reperfusion injury (Novgorodov et al., 2016). In contrast, another study showed that SIRT3 inhibition did not exert a neuroprotective effect after I/R injury *via* the SIRT3 pathway (Verma et al., 2019). Further, another study found that SIRT3 could reduce cerebral I/R injury by regulating the Wnt/beta-catenin pathway and inhibiting mitochondrial fission (Zhao et al., 2018). Most current studies have shown that the neuroprotective function of SIRT3 is associated with the modulation of mitochondrial function, antioxidative stress, anti-inflammatory and autophagy-related pathways, and programmed cell death (Tyagi et al., 2018; Yang X. et al., 2021; Li Y. et al., 2021; Hei et al., 2022). Recent studies have found that modulating the tunneling nanotubes formation and transcellular mitochondria transfer is a promising strategy for the treatment of ischemic stroke (Luchetti et al., 2022), however, the relationship between Sirt3 and tunneling nanotubes formation and transcellular mitochondria transfer remains unclear. Changes in the blood-brain barrier directly caused by endothelial injury play a significant role in the pathogenesis of ischaemic stroke (Yang X. et al., 2021), and SIRT3 plays a critical role in alleviating vascular endothelial injury after stroke (Liu et al., 2022). In addition, SIRT3 can aid neurovascular recovery in an ischaemic stroke model (Yang X. et al., 2018).

## 5 The role of SIRT3 in SAH

In a study by Huang et al., it was found that SIRT3 was highly expressed in normal cerebral cortical neurones and endothelial cells but was rarely expressed in glial cells (Huang et al., 2016). SIRT3 plays an essential neuroprotective role in subarachnoid haemorrhage; however, changes in SOD2, an important downstream molecule of SIRT3, tend to vary according to the current research. In early brain injury after carotid artery puncture in rats with subarachnoid haemorrhage, SIRT3 levels decreased with increasing ROS (Huang et al., 2016). The results showed that SIRT3 mRNA and protein expression decreased significantly 8 h after SAH and decreased to their lowest levels at 24 h. There is evidence that the expression of SOD2 increases after subarachnoid haemorrhage and climbs to the top at 24 h because of neuroinflammation and neuronal apoptosis (Yang S. et al., 2018). However, subsequent research revealed that the levels of both SIRT3 and SOD2 decreased following subarachnoid haemorrhage (Zhang Y. et al., 2019; Zhang K. et al., 2020; Chen T. et al., 2022). To explore the protective role of SIRT3 in early brain injury after subarachnoid haemorrhage, researchers have focused on the regulation of mitochondrial homeostasis by SIRT3, which is not limited to non-inflammation and apoptosis. Currently, most studies have focused on the role of SIRT3 in early brain injury (Wu X et al., 2020; Chen et al., 2023). However, there are few studies on delayed cerebral ischaemia and late complications following SAH, which further indicates that early brain injury plays a crucial role in promoting the development of pathophysiological mechanisms after subarachnoid haemorrhage.

## 6 The role of SIRT3 in TBI

In one study, SIRT3 expression was shown to be enhanced 24 h after TBI *via* a small-molecule antioxidant that could further elevate SIRT3 levels to exert neuroprotective effects (Wang et al., 2016). However, another study demonstrated that the expression of SIRT3 in the TBI group was lower than that in the sham group (Chen T. et al., 2021). Current research shows that SIRT3 plays an important role in brain protection through diverse mechanisms, including maintenance of mitochondrial function and alleviation of blood-brain barrier destruction after TBI. In recent years, the role of ferroptosis in brain injury has attracted considerable attention (Novgorodov et al., 2018). Studies have shown that SIRT1 is involved in neuronal ferroptosis after SAH (Yuan et al., 2022). However, the role of the SIRT1-SIRT3 axis in ferroptosis after brain injury requires further investigation. Additionally, SIRT3 knockdown aggravates nerve cell necrosis, which is not conducive to cell survival following glutamate-induced nerve cell damage. (Novgorodov et al., 2018). This shows that SIRT3 plays a significant role in alleviating glutamate-induced neuronal necrosis; however, the relationship between SIRT3 and neuronal necrosis after TBI remains to be elucidated. Adiponectin (APN), a 30 kDa adipocytokine, has been found to protect neurological function following traumatic brain injury, and PRDX3, a critical antioxidant enzyme in mitochondria, has been identified as a significant downstream target of the APN/SIRT3 axis to reduce oxidative damage following TBI (Zhang S. et al., 2022). Current studies of SIRT3 in TBI have focused on several mechanisms involved in mitochondrial oxidative damage and apoptosis; however, the mechanisms by which SIRT3 is associated with mitochondrial dysfunction, such as mitochondrial dynamics and mitochondrial biogenesis, require further investigation.

## 7 The potential therapeutic agents modulating SIRT3

Many factors influence SIRT3 expression and activity, including drugs, compounds, natural extracts, peptides, physical stimulation, and other small molecules. SIRT3 modulation is affected by drugs such as perampanel, melatonin, metformin, dexmedetomidine, and gastrodin; natural extracts, namely, trilonbatin, piceatannol, resveratrol, and Icariside II; compounds include stilbene and phenolic compounds such as sesamin, sesamol, and 5-heptadecylresorcinol; peptides such as Elabela, a novel kidney-protective peptide CHBP; and physical stimuli that include mild hypothermia, ionising radiation (IF), and electrical stimulations. In addition, physical exercise has been shown to upregulate SIRT3 expression (Figure 2).

Melatonin plays a vital role in haemorrhagic and ischaemic strokes *via* the SIRT3 pathway (Yang S. et al., 2018; Liu L. et al., 2019; Liu L. et al., 2021). Whether it exerts its effects through SIRT3 in TBI or other types of brain injury requires further exploration. An experimental model of stroke demonstrated the protective role of perampanel against blood-brain barrier disruption (Niu et al., 2018; Kawakita et al., 2022). The activation of microglia and production of proinflammatory mediators play essential roles in different types of brain injury. Therefore, regulating microglial activation is a potential therapeutic strategy to ameliorate brain damage (Liu S. J. et al., 2018). Gastrodin, an herbal remedy with neuroprotective properties, can reduce the generation of proinflammatory mediators in activated microglia *via* the SIRT3 pathway (Guo et al., 2021). *In vitro*, sesamin and sesamol chemicals produced from sesame seeds and oil shield neuronal cells against apoptosis and act as antioxidants. Additionally, the activation of SIRT1-SIRT3-FOXO3a expression is linked to the molecular mechanisms that enable the neuroprotection of these chemicals (Ruankham et al., 2021). Further, phenolic substances exhibit neuroprotective properties *in vitro* (Liu et al., 2020). Thus, future studies involving *in vivo* neuroprotective models are required to evaluate these antioxidants. Similar to apelin, which affects body fluid balance, heart health, and renal insufficiency, Elabela is a novel peptide that binds to the apelin receptor. In diabetic rats, Elabela controls oxidative stress *via* the SIRT3-Foxo3a pathway to avoid myocardial injury (Li C. et al., 2021), and its role in preventing burn injury merits further study. Similarly, a new polypeptide has been discovered with neuroprotective effects (Wang et al., 2017). Trilobatin, a natural agonist, protects neurones from oxidative stress by modulating mtROS homeostasis, which is mediated in part by the AMPK/Nrf2/SIRT3 signalling pathway (Gao et al., 2018b). Icariside II activates the Nrf2/SIRT3 signalling pathway, which reduces oxygen-glucose deprivation and reoxygenation-induced PC12 cell oxidative damage (Feng et al., 2018). However, these results need to be confirmed *in vivo*. The results provide novel insights into how piceatannol could be used as a promising bioactive component against oxidative damage and neurocyte apoptosis, mainly involving the SIRT3 pathway (Yang et al., 2022).

Ionising radiation increases the expression and enzyme activity of SIRT3 (Richardson et al., 2022). However, the safety of ionising radiation limits the regulation of SIRT3 in the brain. The therapeutic effects of electrical stimulation on posttraumatic neurological dysfunction and disturbance of consciousness have been widely studied. However, it is unclear whether regulation of SIRT3 by electrical stimulation is feasible (Cong et al., 2020). Hydrogen sulphide is a gas transmitter with various cardiovascular protective effects (Zhang et al., 2018). Studies have shown that exogenous supplementation with hydrogen sulphide can combat SIRT3-dependent antioxidant stress in mice with cardiac hypertrophy. Nevertheless, it is unclear whether hydrogen sulphide could exert a role in haemorrhage or ischaemic stroke. There is insufficient evidence to indicate an association between the SIRT3 pathway and the clinical application of mild hypothermia to treat patients in the early stages of SAH and TBI, which exerts a protective role by rescuing mitochondrial dysfunction. Energy restriction can regulate the expression of SIRT3 (Zhang J. et al., 2019). However, studies have shown that energy restriction attenuates ischaemia-reperfusion injury not through the SIRT3 pathway but by professional adiponectin, which plays a protective role in TBI through the SIRT3 pathway (Zhang J. et al., 2019; Liu H. et al., 2020; Zhang S. et al., 2022). It is unclear whether caloric restriction acts *via* the SIRT3 pathway to protect against other types of brain injury, such as subarachnoid haemorrhage. Luteolin may protect against cerebral I/R damage by controlling the SIRT3/AMPK/mTOR signalling pathway (Liu S. et al., 2020). Crocin-I can alleviate neuroinflammation and oxidative stress in the mouse hippocampus and restore mitochondrial function in a depression model (Xiao et al., 2019).

## 8 Conclusion

SIRT3, a member of the sirtuin family found in the mitochondria, plays an essential role in body injury or stress. Most studies have focused on the acute processes that occur after several types of brain damage. The long-term effects of targeting SIRT3, as well as the dynamic alteration of SIRT3 after brain injury, require further exploration. In addition, the effects on other members of the SIRT family, such as SIRT6 and SIRT7, and the relationship between SIRT3 and other members of the SIRT family are not known. Several factors, including molecules or drugs that modulate SIRT3, can attenuate brain injury. However, the safety and toxicity, absorption and metabolic rate of drugs, and permeability of the blood-brain barrier are still worthy of consideration. Further investigations are required to realise clinical translation and drug exploitation.

Moreover, evidence is still lacking regarding the two-sided effect of SIRT3 in other types of brain injuries, such as subarachnoid haemorrhage, TBI, and intracerebral haemorrhage. However, SIRT3 shows different changes in TBI, which may be related to the experimental model and the environment. Whether SIRT3 can interact with and affect other organelles in the cell, including the endoplasmic reticulum, requires further investigation. Currently, most data come from cellular and animal experiments, and most functions of SIRT3 have yet to be verified in neuronal cells. Therefore, to overcome the challenges of clinical transformation of the present literature and to verify the brain-protective effect of SIRT3, extensively researched clinical evidence is required.
